# Neurobeachin, a Regulator of Synaptic Protein Targeting, Is Associated with Body Fat Mass and Feeding Behavior in Mice and Body-Mass Index in Humans

**DOI:** 10.1371/journal.pgen.1002568

**Published:** 2012-03-15

**Authors:** Pawel K. Olszewski, Jan Rozman, Josefin A. Jacobsson, Birgit Rathkolb, Siv Strömberg, Wolfgang Hans, Anica Klockars, Johan Alsiö, Ulf Risérus, Lore Becker, Sabine M. Hölter, Ralf Elvert, Nicole Ehrhardt, Valérie Gailus-Durner, Helmut Fuchs, Robert Fredriksson, Eckhard Wolf, Thomas Klopstock, Wolfgang Wurst, Allen S. Levine, Claude Marcus, Martin Hrabě de Angelis, Martin Klingenspor, Helgi B. Schiöth, Manfred W. Kilimann

**Affiliations:** 1 Functional Pharmacology Unit, Department of Neuroscience, Uppsala University, Uppsala, Sweden; 2 Minnesota Obesity Center, University of Minnesota, St. Paul, Minnesota, United States of America; 3 German Mouse Clinic, Institute of Experimental Genetics, Helmholtz Zentrum München, German Research Center for Environmental Health, Neuherberg, Germany; 4 Molecular Nutritional Medicine, Else Kröner-Fresenius Center and ZIEL Research Center for Nutrition and Food Sciences, Technische Universität München, Freising-Weihenstephan, Germany; 5 Chair for Molecular Animal Breeding and Biotechnology, Gene Center, Ludwig-Maximilians-Universität München, München, Germany; 6 Molecular Cell Biology Unit, Department of Neuroscience, Uppsala University, Uppsala, Sweden; 7 Department of Public Health and Caring Sciences, Uppsala University, Uppsala, Sweden; 8 Department of Neurology, Friedrich-Baur-Institut, Ludwig-Maximilians-Universität München, München, Germany; 9 Helmholtz Zentrum München, German Research Center for Environmental Health, Institute of Developmental Genetics, Neuherberg, Germany; 10 Max-Planck-Institute for Psychiatry, München, Germany; 11 Technical University München-Weihenstephan, Lehrstuhl für Entwicklungsgenetik c/o Helmholtz Zentrum München, Neuherberg, Germany; 12 Deutsches Zentrum für Neurodegenerative Erkrankungen (DZNE) Munich, Germany; 13 Department for Clinical Science, Intervention, and Technology, Karolinska Institutet, Division of Pediatrics, National Childhood Obesity Centre, Stockholm, Sweden; 14 Lehrstuhl für Experimentelle Genetik, Technische Universität München, Freising-Weihenstephan, Germany; University of Cincinnati, United States of America

## Abstract

Neurobeachin (Nbea) regulates neuronal membrane protein trafficking and is required for the development and functioning of central and neuromuscular synapses. In homozygous knockout (KO) mice, Nbea deficiency causes perinatal death. Here, we report that heterozygous KO mice haploinsufficient for Nbea have higher body weight due to increased adipose tissue mass. In several feeding paradigms, heterozygous KO mice consumed more food than wild-type (WT) controls, and this consumption was primarily driven by calories rather than palatability. Expression analysis of feeding-related genes in the hypothalamus and brainstem with real-time PCR showed differential expression of a subset of neuropeptide or neuropeptide receptor mRNAs between WT and *Nbea*+/− mice in the sated state and in response to food deprivation, but not to feeding reward. In humans, we identified two intronic *NBEA* single-nucleotide polymorphisms (SNPs) that are significantly associated with body-mass index (BMI) in adult and juvenile cohorts. Overall, data obtained in mice and humans suggest that variation of Nbea abundance or activity critically affects body weight, presumably by influencing the activity of feeding-related neural circuits. Our study emphasizes the importance of neural mechanisms in body weight control and points out *NBEA* as a potential risk gene in human obesity.

## Author Summary

Body weight and energy balance are under very complex neural, endocrine, and metabolic control. Correspondingly, recent research suggests that hundreds of genes contribute to human obesity and that only a small proportion of them have as yet been identified. Neurobeachin (Nbea) is a protein specifically expressed in nerve and endocrine cells and is important for neurotransmission, apparently by influencing the synaptic targeting of membrane proteins. Here, we show that heterozygous knockout mice, expressing Nbea at 50% of normal levels, display increased adipose tissue mass, abnormal feeding behavior, and modified expression of specific genes in the brainstem and hypothalamus known to be important for body weight control. Moreover, we find that *NBEA* gene polymorphisms are associated with body-mass index in adult and juvenile human cohorts. Our results demonstrate that variation of Nbea activity critically affects body weight, presumably by influencing the activity of feeding-related neural circuits. They emphasize the importance of neural mechanisms in body weight control, and they identify *NBEA* as a potential genetic risk factor in human obesity.

## Introduction

The BEACH (*be*ige *a*nd *Ch*ediak-Higashi) domain protein family is implicated in the intracellular targeting of membrane proteins. Its members have been found in yeasts, amoebas, plants and animals, suggesting involvement in fundamental cellular functions. Mutations in BEACH domain proteins result in complex defects of cellular membrane dynamics and membrane protein targeting [1]–[5].

One of the eight mammalian BEACH proteins [5] is Neurobeachin (Nbea), a 327-kDa molecule expressed in neurons and endocrine cells. Nbea contains a high-affinity binding site for the type II regulatory subunit of protein kinase A (PKA) [6], which classifies it as an A-kinase anchor protein (AKAP). AKAPs anchor and concentrate the PKA holoenzyme at defined subcellular locations, enhancing the efficiency and specificity of the interaction of PKA with selected subsets of its target proteins [7].

Nbea is associated with polymorphic vesiculo-tubulo-cisternal endomembranes and postsynaptic plasma membranes, and it is found at high concentrations near the *trans* side of Golgi stacks. Therefore, a role of Nbea in the post-Golgi sorting or targeting of membrane proteins was proposed [6]. Nbea is essential for synaptic neurotransmission at neuromuscular junctions (NMJ): Nbea-null mice generated via coincidental insertion mutagenesis die immediately after birth due to breathing paralysis caused by a complete block of evoked transmission at NMJs [8]. In independently derived Nbea KO mice, central neurons showed impaired neurotransmission at both excitatory and inhibitory synapses, lower synapse density and altered synaptic protein composition while the lethal NMJ phenotype was also confirmed. The electrophysiological phenomena at central synapses suggested defects of both presynaptic neurotransmitter release and postsynaptic response, e.g., through reduced neurotransmitter receptor density [9].

Human data on NBEA are very limited, but heterozygous disruptions in the *NBEA* gene have been linked with autism and multiple myeloma. A *de novo* translocation in the *NBEA* gene was detected in an autistic patient [10], and additional evidence linking deletions of the chromosomal region containing *NBEA* to autism has been found ([11]; OMIM 608049). Heterozygous deletions involving *NBEA* were found in a subgroup of multiple myeloma patients [12], and *NBEA* was shown to harbor a region of enhanced chromosomal fragility [11], [13].

Homozygous inactivation of the *Nbea* gene in mice results in perinatal death, whereas heterozygous *Nbea* KO mice are viable and fertile and do not display obvious abnormalities. The association of heterozygous human *NBEA* mutations with autism and cancer suggested that NBEA haploinsufficiency may produce related phenotypes in mice, and we therefore investigated *Nbea*+/− mice in the phenotyping screen of the German Mouse Clinic (GMC). While the possible involvement of Nbea in autism and cancer requires further study, we unexpectedly found phenotypic features of these mice implicating Nbea in energy balance regulation: significantly greater body weight and adipose tissue mass and an elevated energy surplus during early life. Subsequently, we detected alterations in feeding behavior of *Nbea*+/− mice in several functional tests investigating the effects of high caloric and highly palatable diets, and in the expression of feeding-related genes in the hypothalamus. Finally, we detected the association of two intronic *NBEA* single-nucleotide polymorphisms (SNPs) with weight and body mass index (BMI) in humans, suggesting that variability within the *NBEA* gene may be a genetic risk factor in human obesity.

## Results

### Haploinsufficient *Nbea*+/− mice on standard chow display faster increase in body weight due to moderately elevated adiposity

**Figure 1:**
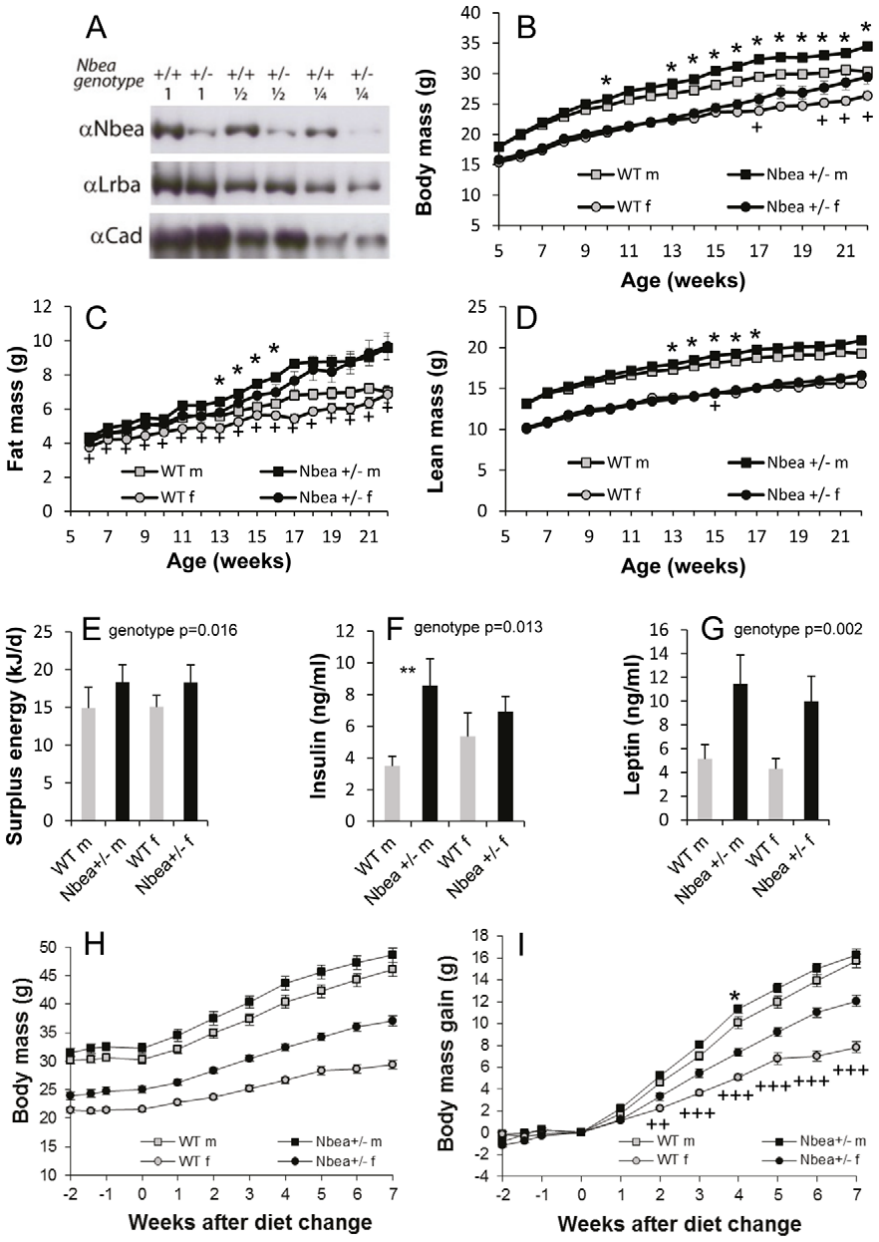
Nbea-haploinsufficient mice develop higher body weight due to higher adipose tissue mass. (A) Western blot analysis of whole brain demonstrates that Nbea protein expression in *Nbea*+/− mice is half of that in WT mice. The same blot was sequentially developed with anti-Nbea, anti-Lrba and anti-Cadherin. 1, ½ and ¼ indicate a dilution series of loaded protein. (B) Male and female *Nbea*+/− mice develop higher body mass than WT controls. From week 14 on we continuously detected a significant genotype effect on body mass in males and females combined (week 14–15 p<0.05, week 16–19 p<0.01, afterwards p<0.001). (C,D) qNMR scans of mice aged 6–22 weeks shows that increased body mass of *Nbea*+/− mice is caused by increased fat tissue mass (significant genotype effect in females between week 6–21, in males in week 13–16, linear model with body mass as covariate). (E) The in-out-difference between daily metabolizable energy and daily energy expenditure was significantly increased in *Nbea*+/− mice at the age of 8 weeks. (F) Plasma insulin and *(G)* leptin at 22 weeks of age were significantly increased in *Nbea*+/− mice. (H,I) High-fat feeding from age 14 weeks accelerates weight gain, more pronouncedly in *Nbea*+/− than in WT mice. * within males, ^+^within females, P<0.05; ** within males, ^++^within females, P<0.01; *** within males, ^+++^within females, P<0.001; error bars, ± SEM. In part *H*, all genotype differences were significant for males with at least P<0.05 (except weeks −2 and 7, n.s.) and for females with at least P<0.001 (except week −2, p<0.01).

The *Nbea* gene-trap KO allele has been described [9]. Mice heterozygous for this allele are viable and fertile and display no obvious abnormalities in observation up to an age of 2 years. We did not observe the dwarfism described by Su et al. [8] for their *Nbea*+/− mice. This phenotypic aspect of the mutants of Su et al. may be due to the specific nature of their mutation (antisense-oriented insertion of a growth hormone minigene). Immunoblot analysis of brain homogenates showed that Nbea protein expression in *Nbea*+/− mice was ∼50% of wild-type (WT) mice (42±6% [mean±SEM], n = 12) whereas the expression level of the Nbea isoform, Lrba, was unaffected (Figure 1A).

*Nbea*+/− mice were systematically analyzed for genotype effects in the primary phenotyping screen at the GMC. Mutant and control mice entered the screen at an age of 9 weeks and were consecutively investigated in the behavior, neurology, dysmorphology, clinical chemistry and energy metabolism screen, among others [14]–[15]. Both male and female mutant mice were slightly but significantly heavier than controls. Dual-energy X-ray absorptiometry (DXA) performed on 16 weeks old WT and *Nbea*+/− mice revealed that the difference in body weight was due to increased body fat content (Table 1). This increased adiposity was apparent in both females and males.

**Table 1:** First-line phenotyping in the German Mouse Clinic: Body composition (DXA) and energy assimilation parameters of 18–20 week-old WT and *Nbea*+/− mice, fed with standard chow *ad libitum* over 5 days (n = 7/group for energy metabolism, n = 15/group for DXA; means±SD).

Parameter	Males		Females		*P* value genotype
	WT	Nbea+/−	WT	Nbea+/−	
Initial body mass (g)	30.8±0.8	32.2±0.9	23.2±0.7	25.9±1.0	<0.05
Body fat mass (g)	6.7±0.9	9.2±1.9	3.7±0.9	7.3±1.0	<0.05*
Lean mass (g)	20.9±1.1	19.8±1.5	16.6±0.7	15.03±0.5	0.05*
Food intake (g/day)	3.5±0.5	4.1±0.4	3.2±0.4	3.3±0.3	n.s.*
Feces production (g/day)	0.75±0.12	0.86±0.12	0.67±0.08	0.73±0.07	<0.05**
Energy content of feces (kJ/g)	15.55±0.09	15.30±0.21	15.47±0.12	15.44±0.12	<0.05
Daily metabolized energy (kJ)	49.5±7.6	59.1±5.7	45.7±7.4	46.1±4.9	n.s.*
Food assimilation coefficient (%)	79.5±0.7	80.5±1.4	79.9±2.8	79.0±0.8	n.s.
Rectal body temp. (°C)	35.75±0.14	35.95±0.23	36.45±0.27	36.46±0.14	0.199

*: Fat and lean mass, food intake and daily metabolized energy were analyzed using a linear model including body mass as covariate.

**: Feces production was analyzed using a linear model including food intake as covariate to adjust for differences in overall food consumption.

As part of the first-line phenotyping screen we then determined food intake and efficiency of energy extraction from the diet in cohorts of 7 mice over 5 days at the age of 18–20 weeks, with *ad libitum* access to standard chow (Table 1). At this stage food intake was higher in *Nbea*+/− mice (p<0.05) but proportional to body mass; if initial body mass was included as a covariate, the statistical analysis detected no genotype effect on food intake. The overall efficiency of energy extraction from food (food assimilation coefficient) did not differ between genotypes. Daily metabolized energy intake was indistinguishable between the two genotypes when adjusted for body weight.

Behavioral analysis of spontaneous activity in a novel environment, measured by the modified Hole Board test at age 8–9 weeks, and neurological analysis according to a modified SHIRPA protocol at age 9–10 weeks, did not indicate reduced spontaneous activity that could cause lower energy expense of the *Nbea*+/− mice. In particular, no reductions of motor activity parameters (e.g., total distance moved) were detected. The only genotype-related abnormality was a slight increase of mean locomotion velocity in both sexes by an average 6.5% (genotype effect, p<0.05), which may indicate a minor perturbation of the locomotor rhythm generator and would, if at all, cause an increased energy expense.

Blood chemical parameters determined at the ages of 12–13 and 17–18 weeks yielded a slightly increased α-amylase activity in both sexes by an average 8% as the only parameter significantly (p<0.05) and reproducibly abnormal in the mutants (age 12–13 weeks: males, WT 2640±90 vs. *Nbea*+/−2790±60; females, WT 1940±50 vs. *Nbea*+/− 2130±40; in U/L±SEM). Sodium, potassium, calcium, chloride, inorganic phosphate, creatinine, triglycerides, cholesterol, urea, uric acid, glucose, total protein, creatine kinase, alanine aminotransferase, aspartate aminotransferase, alkaline phosphatase, ferritin, transferrin and lipase were unaffected by genotype.

In a second cohort of mice we monitored body mass and body composition weekly during early lifetime. We could confirm the development of mild obesity both in *Nbea*+/− males and females (Figure 1B–1D). To evaluate daily energy balance, we monitored both sides of the energy balance equation, i.e. food intake and energy assimilation and energy expenditure, by gas exchange measurements over 24 hours at eight weeks of age. Indirect calorimetry did not reveal statistically significant differences in daily energy expenditure (Table 2). Monitoring of food intake and bomb calorimetry of feces and diet samples to determine caloric uptake and the amount of metabolizable energy indicated that energy uptake was slightly increased in mutant animals but the difference did not reach statistical significance when analyzed by a linear regression model including genotype, sex and body mass (Table 2). Calculating the difference between daily metabolizable energy and daily energy expenditure showed that both WT and *Nbea*+/− mice were in a positive energy balance at 8 weeks of age (Table 2 and Figure 1E). In WT mice this surplus of energy, expressed as in-out difference in Table 2, was in the range of ∼15 kJ per day reflecting the normal energy demand for growth in 8 weeks old mice. Notably, the surplus of energy in *Nbea*+/− mice was slightly higher with ∼18 kJ per day. When compared to WT mice, *Nbea*+/− mice had 3.2 kJ (females) and 3.4 kJ (males) excess energy available on a per day basis (Table 2). This effect of genotype was significant when body mass changes during the indirect calorimetry trial were included in the linear regression model. Continuous monitoring of spontaneous motor activity (distance traveled, rearing) and determination of body temperatures again ruled out both parameters as explanations for the increased fat mass and positive energy balance of *Nbea*+/− mice (Table 2). Plasma insulin (males +146%, females +29%, genotype p = 0.013) and leptin levels (males +122%, females +131%, genotype p = 0.002), determined at 22 weeks of age, were significantly increased in *Nbea*+/− mice (Figure 1F, 1G). When adjusted for body fat content, however, no difference in leptin levels could be detected. Resistin and PAI-1 were not different between genotypes.

**Table 2:** Energy balance and motor activity of 8 week-old WT and *Nbea*+/− mice, fed with standard chow *ad libitum* over 24 h (n = 8/group; mean±SD).

Parameter	Males		Females		*P* value genotype
	WT	Nbea+/−	WT	Nbea+/−	
Body mass (g)	23.7±1.3	24.1±1.3	19.2±1.6	19.9±2.6	n.s.
Food intake (g/day)	4.7±0.6	5.0±0.5	4.5±0.4	4.7±0.7	n.s.*
Daily metabolized energy (kJ)	60.6±7.9	65.4±6.5	58.1±4.8	61.2±8.6	n.s.*
Oxygen consumption (ml/h)	91.4±3.7	94.0±4.8	85.7±0.6	85.3±5.8	n.s.*
Respiratory exchange ratio	0.94±0.02	0.95±0.01	0.95±0.01	0.97±0.03	n.s.
Daily energy expenditure (kJ)	45.7±1.9	47.1±2.3	43.0±4.3	42.9±3.0	n.s.*
In-out difference (kJ)	14.9±7.8	18.3±6.7	15.1±4.3	18.3±6.7	0.016**
Distance travelled (m/d)	398±87	385±88	570±252	450±152	n.s.
Rearing activity (counts/d)	7010±2242	6177±2510	9500±4174	11153±13813	n.s.
Rectal body temp. (°C)	35.8±0.4	36.1±0.4	36.1±0.2	36.4±0.2	0.068

*: Food intake, daily metabolized energy intake, oxygen consumption and daily energy expenditure were analyzed using a linear model including body mass as covariate accounting for the effects of body mass differences on energy metabolism parameters.

**: In-out difference was analyzed using a linear model including weight changes during the indirect calorimetry trial as covariate.

Several mouse models for obesity exhibit normal or only slightly increased body weight on standard diets but develop increased adiposity in response to high-fat (HF) diet intake [16]. Therefore, *Nbea*+/− and WT mice were fed a HF diet (60 energy% fat) from the age of 14 weeks. In this third cohort of mice, *Nbea*+/− males and females weighed, respectively, 2.0 g and 3.6 g more than WT mice at the onset of HF feeding, again confirming the body mass phenotype. In response to HF feeding, *Nbea*+/− females, in particular, exhibited increased susceptibility to diet-induced obesity. After 7 weeks of HF feeding *Nbea*+/− females gained substantially more weight than WT (4.1 g), whereas the differential weight gain of *Nbea*+/− males in excess of WT was marginal (0.6 g) (Figure 1H, 1I). A glucose tolerance test after 22 weeks revealed no genotype effects on baseline glucose level or glucose response, in males or females (not shown).

### *Nbea*+/− mice overeat when motivated by hunger or by the diet's incentive combination of energy and palatability, but not by palatability alone

**Figure 2:**
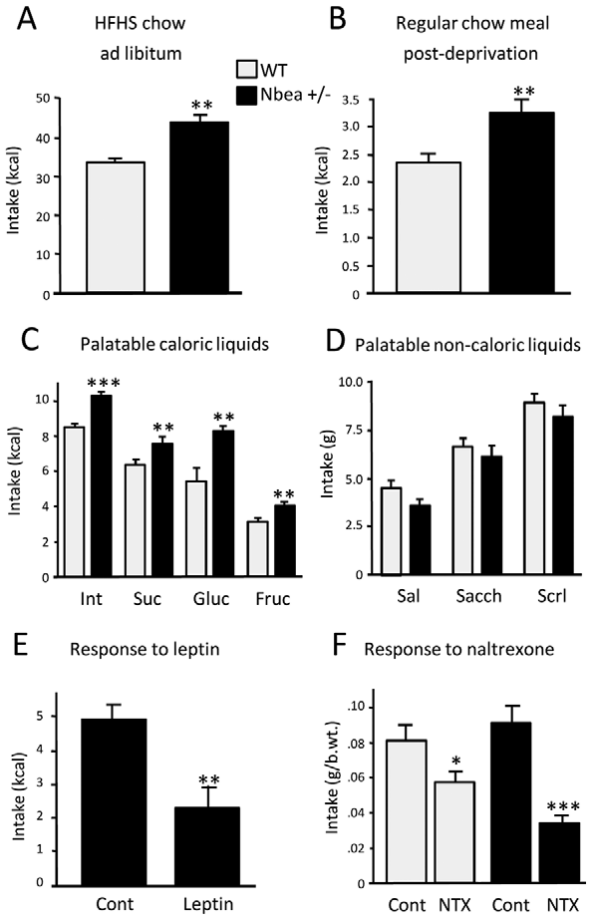
Differential feeding behavior of *Nbea+/−* and WT mice. Male mice were tested at the age of 8–10 weeks, prior to the manifestation of a significant body weight difference between the genotypes. Compared to WT mice, *Nbea*+/− mice *(A)* ate more *ad libitum* of the energy-dense and palatable high-fat high-sugar (HFHS) solid diet; *(B)* ate more standard chow upon 2-h refeeding after overnight food deprivation; *(C)* consumed more of the caloric and palatable 4.1% Intralipid, 10% sucrose, 10% glucose and 10% fructose solutions; but *(D)* they consumed the same amounts of tastants which do not contain calories, i.e. saline, 0.1% saccharin or 0.05% sucralose, despite their palatability. *Nbea*+/− mice *(E)* were not resistant to leptin, but they were *(F)* more sensitive to the anorexigenic naltrexone (NTX) than their WT counterparts. *, P<0.05; **, P<0.01; ***, P<0.001; error bars, ± SEM.

As the previous experiments detected mild differences in gross energy balance between *Nbea*+/− and WT mice, we tested for more subtle modifications of feeding behavior in episodic feeding paradigms. During these tests, male mice were between the 8^th^ and 10^th^ week of age, before the emergence of a statistically significant body weight difference between the genotypes. Although, at this age, *Nbea*+/− mice did not consume detectably more standard chow when fed *ad libitum* (*Nbea*+/−: 174.8±1.0 g/kg body weight; WT: 177±2.3 g/kg body weight), the combined incentive of high calorie content and palatability of a high-fat/high-sugar (HFHS) diet stimulated them to eat significantly more than WT controls (Figure 2A). When refed after overnight food deprivation, *Nbea*+/− mice ingested more also of the standard chow (Figure 2B). Similarly, daily consumption of caloric palatable fluids (Intralipid fat emulsion, sucrose, glucose, fructose) was higher in *Nbea*+/− than in WT mice (Figure 2C). In contrast, non-caloric yet palatable tastants (saline, saccharin, sucralose) were not overconsumed by the *Nbea*+/− animals (Figure 2D).

### *Nbea*+/− mice are leptin-responsive and naltrexone-hyperresponsive

Obesity may be caused by leptin resistance. Resistant animals injected with leptin prior to a meal do not reduce food consumption. When we treated overnight-deprived *Nbea*+/− mice with leptin at the time of chow refeeding, they ate significantly less food than control animals receiving only saline injection (Figure 2E), indicating that leptin resistance is unlikely to explain the obesity of *Nbea*+/− mice. Resistance to leptin is also marked by the lack of responsiveness of hypothalamic neurons, which relay the effects of leptin at the central level, to hormone infusion. A preliminary c-Fos induction experiment (Figure S1) showed that leptin infusion increased the density of Fos-immunopositive, activated neurons in the arcuate nucleus (ARC) of an *Nbea*+/− and a WT mouse alike, confirming that the mutant mice are sensitive to leptin.

An anorexigenic dose of the opioid receptor antagonist, naltrexone (NTX), administered peripherally to overnight-deprived mice just prior to chow refeeding, caused a ∼30% reduction in food intake in WT animals. *Nbea*+/− mice consumed ∼60% less food than saline controls (Figure 2F), showing that they are responsive to NTX, indeed even hyperresponsive (p = 0.04).

### Hypothalamic expression of genes involved in feeding regulation differs between *Nbea*+/− and WT mice in the baseline sated state and in the hungry state, but not upon intake of palatable foods

**Figure 3:**
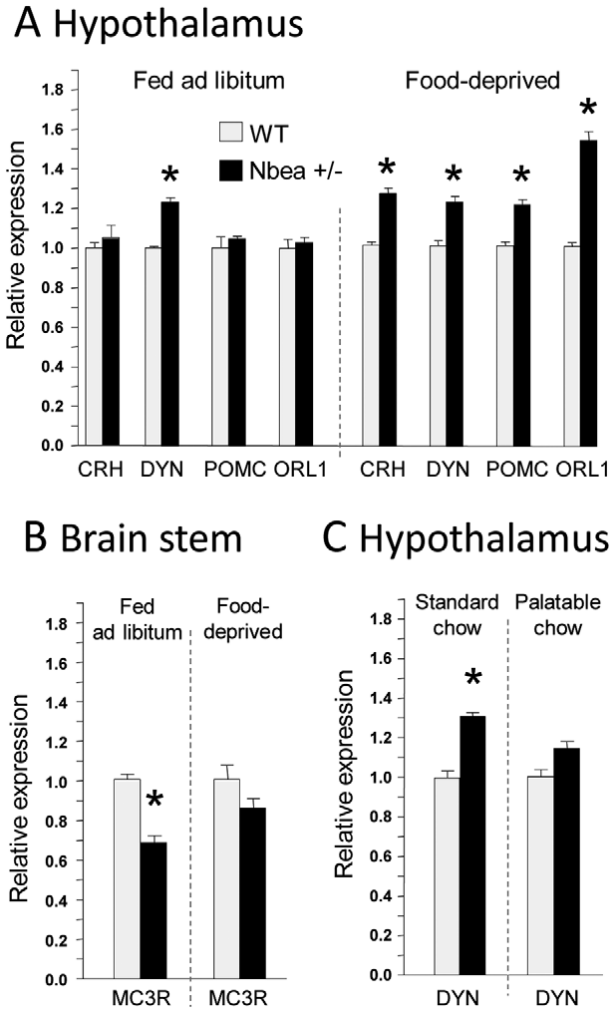
Differential expression of feeding-related genes in *Nbea+/−* and WT mice under different feeding paradigms. *Ad libitum*-fed, sated *Nbea*+/− and WT mice showed a differential expression of the dynorphin (DYN) mRNA in the hypothalamus *(A)* and of the melanocortin (MC) receptor-3 mRNA in the brainstem *(B)*. 16-h food deprivation differently affected expression of mRNAs encoding DYN, proopiomelanocortin (POMC), opioid-like receptor-1 (ORL1) and corticotropin releasing hormone (CRH) in the hypothalamus *(A)*, whereas no differences between the genotypes were triggered by food deprivation in the brainstem *(B)*. Exposure to the palatable HFHS diet did not affect gene expression differently in the *Nbea*+/− and WT animals. Only DYN mRNA was increased in the standard chow-fed controls *(C)*. * P<0.05; error bars, ± SEM. The following other genes were also analyzed, but their expression levels did not differ between the genotypes: AGRP, Agouti-related protein; AVP, vasopressin; CCK, cholecystokinin; CRHR, CRH receptor; DOR, delta opioid receptor; ENK, enkephalin; GHSR, growth hormone secretagogue receptor; GLP1R1, glucagon-like peptide 1 receptor 1; KOR, kappa opioid receptor; MCH, melanin concentrating hormone; MOR, mu opioid receptor; NPY, neuropeptide Y; ORX, orexin.

Following the identification of altered feeding behavior described above, we hypothesized that reduced Nbea expression may alter the activity of neuronal networks involved in energy balance control. We investigated whether expression of feeding-related genes differs between the *Nbea*+/− and WT genotypes in response to different food availability/quality regimens: in *ad libitum* feeding of standard chow, following food deprivation, and when a palatable diet is offered. *Ad libitum*-fed *Nbea*+/− mice expressed a higher level of orexigenic dynorphin (DYN) mRNA compared to WT controls in the hypothalamus (Figure 3A). Moderate-length (16 h) food deprivation led to higher mRNA expression of four hypothalamic genes in *Nbea*+/− vs. WT mice. Three of them: DYN, proopiomelanocortin (POMC) and opioid-like receptor-1 (ORL1), are linked with orexigenic responses (POMC gives rise to orexigenic beta-endorphin, but also to hypophagic melanocortins (MC)), whereas corticotropin releasing hormone (CRH) is involved in the HPA axial activity and satiety signaling. In the brainstem, only the MC3 receptor mRNA level was lower in *Nbea*+/− than in WT mice in the *ad libitum* paradigm, and none of the markers were differentially affected by food deprivation (Figure 3B). Animals exposed to the palatable diet did not show any difference between genotypes in their gene expression response in the hypothalamus (Figure 3C).

### *NBEA* gene polymorphism is associated with body-mass index (BMI) in human cohorts

We then examined whether the link between Nbea and body weight control identified in the mouse model can be extended to humans. We studied the association of two relatively common SNPs with body weight and BMI. Two intronic SNPs in *NBEA*, rs17775456 and rs7990537 (r^2^ = 0.12), were genotyped in two cohorts: one comprising adult men from the ULSAM cohort and one case-control cohort of children and adolescents. Table 3 shows anthropometric characteristics in the two cohorts according to *NBEA* rs17775456 and rs7990537 genotype and weight status. None of the two SNPs caused deviation from Hardy-Weinberg equilibrium in any of the studied cohorts (p>0.05). We analyzed the subjects for associations with overweight and obesity depending on *NBEA* genotypes (Table 4) as well as BMI and weight as continuous traits (Table 5). We found a significant association for rs17775456 and rs7990537 with BMI as a continuous trait (rs17775456: p = 0.001 and rs7990537: p = 0.006) and trends for weight (rs17775456: p = 0.022 and rs7990537: p = 0.025) among the overweight adult men. Carriers of the minor allele were heavier and had higher BMI than non-carriers. Among children and adolescents, we found that rs7990537 was significantly associated with BMI in the normal-weight children, the carriers of the minor allele again having higher BMI standard deviation scores (SDS).

**Table 3:** Anthropometric characteristics in 50 year-old men and children stratified according to weight status and *NBEA* rs17775456 and rs7990537 genotype.

rs17775456	TT	TA	AA
**Normal weight adult men, n = 597**	2	85	510
Weight (kg)	71.6±6.8	70.3±7.0	67.0±7.1
BMI	22.2±1.7	22.1±1.4	21.0±1.4
**Overweight adult men, n = 497**	2	54	441
Weight (kg)	93.0±18.4	85.6±9.6	84.7±7.8
BMI	29.0±5.7	27.4±2.5	26.7±2.1
**Normal-weight children, n = 520**	3	76	441
Weight (kg)	64.7±10.1	61.7±9.4	63.9±10.0
BMI SDS	0.330±0.581	0.026±0.480	0.173±0.588
**Obese children, n = 499**	3	54	442
Weight (kg)	111.0±42.3	91.6±29.9	91.4±28.9
BMI SDS	7.5±0.7	5.9±1.5	5.7±1.5

rs7990537	GG	GA	AA
**Normal weight adult men, n = 609**	20	211	378
Weight (kg)	71.8±6.9	68.9±9.0	71.1±6.4
BMI	22.1±2.7	21.8±1.9	22.3±1.5
**Overweight adult men, n = 507**	18	146	343
Weight (kg)	87.2±11.1	85.8±8.0	84.1±7.9
BMI	28.0±3.1	27.0±2.1	26.7±2.0
**Normal-weight children, n = 520**	17	179	324
Weight (kg)	65.4±9.2	63.8±10.3	62.9±9.3
BMI SDS	0.183±0.581	0.107±0.577	−0.062±0.442
**Obese children, n = 501**	23	166	312
Weight (kg)	94.7±31.2	89.5±20.0	89.6±28.4
BMI SDS	5.9±1.9	5.7±1.5	5.7±1.4

Data are means ± SD divided into genotype groups.

**Table 4:** Odds ratio for overweight and obesity depending on *NBEA* genotypes in adults and children.

		N	Genotype, n (%)			MAF %	OR (95% CI)	P
Adults			G1	G2	G3		GRR (95% CI)	
**rs17775456**	Normal weight	561	2 (0.4%)	64 (11.4%)	495 (88.2%)	6.0	1.241 (0.887–1.735)	0.207
**A>T**	Overweight	533	2 (0.4%)	75 (14.1%)	456 (85.5%)	7.0	1.22 (0.904–1.669)	
**rs7990537**	Normal weight	574	20 (3.5%)	169 (29.4%)	385 (67.1%)	18.0	1.170 (0.949–1.444)	0.142
**A>G**	Overweight	542	18 (3.3%)	188 (34.7%)	336 (62.0%)	21.0	1.161 (0.991–1.360)	

		N	Genotype, n (%)			MAF %	OR (95% CI)	P
Children			G1	G2	G3		GRR (95% CI)	
**rs17775456**	Normal weight	520	3 (0.6%)	76 (14.6%)	441(84.8%)	7.0	0.747 (0.529–1.055)	0.706
**A>T**	Obese	499	3 (0.6%)	54 (10.8%)	442(88.6%)	6.0	0.752 (0.547–1.033)	
**rs7990537**	Normal weight	520	17 (3.3%)	179 (34.4%)	324 (62.3%)	20.5	1.042 (0.842–1.290)	0.097
**A>G**	Obese	501	23 (3.3%)	166 (34.4%)	312 (62.3%)	21.2	1.000 (0.854–1.172)	

Data are number of subjects in each group and number of subjects for each genotype (G) (% in each group). Allele frequency (A) for each group is given in percentage. GRR indicate genotype relative risk with a 95% confidence interval (CI). Odds ratio (OR) with a 95% confidence interval (CI) was calculated assuming an additive model. Association with overweight in the adult cohort was determined comparing subjects with normal weight (BMI<25 kg/m^2^) and overweight (BMI≥25 kg/m^2^). P indicates p-values adjusted for age and gender.

**Table 5:** Association with BMI and body weight as continuous traits in adults and children for *NBEA* rs17775456 and rs7990537.

		Adult				Children			
SNP	Phenotype	Overweight		Normal-weight		Obese		Normal-weight	
		Beta	P_Add_	Beta	P_Add_	Beta	P_Add_	Beta	P_Add_
**rs17775456**	Body weight (kg)	0.014	0.022	0.013	0.164	0.823	0.487	1.358	0.330
	BMI	0.151	0.001	−0.072	0.208	0.196	0.353	0.188	0.435
**rs7990537**	Body weight (kg)	0.016	0.025	0.015	0.201	0.306	0.198	1.194	0.056
	BMI	0.094	0.006	−0.046	0.216	0.019	0.765	0.208	0.005

Beta indicates transformed beta-values. P indicates p-values adjusted for significant covariates.

## Discussion

For years, obesity research focused primarily on ligand-receptor interactions as the basis of feeding and metabolic responses. Consequently, improper functioning of the ligand-receptor system was considered a causative factor underlying dysregulation of body weight. While this holds true for many such systems, including melanocortins, leptin, ghrelin and neuropeptide Y [17]–[20], it has become clear that molecules which are not directly involved in communication at the cell surface, but are part of intracellular mechanisms, e.g., the nucleic acid demethylase Fto, also play a crucial role in the control of energy homeostasis [21]. Here, we report a combination of mouse and human data pertaining to Nbea, a regulator of membrane protein trafficking, showing that mice heterozygous for the *Nbea* KO allele develop an obese phenotype and that two intronic *NBEA* SNPs are associated with weight and BMI in humans. Nbea is one of a growing number of proteins important for synaptic development and function that is also associated with obesity. Changes in the Nbea status appear to affect select metabolic and feeding-related parameters.

*Nbea*+/− mice develop moderately elevated body weight during early adulthood. Body composition analyses show that this higher body weight stems from increased adipose tissue mass. Lean mass is virtually unaffected (slightly lower in the experiment of Table 1, slightly higher in the experiment of Figure 1D), suggesting altered partitioning of energy in these animals in favor of energy preservation and storage. Therefore, the phenotype of the *Nbea*+/− mouse can be defined as mildly obese. Increased insulin levels are consistent with this phenotype. Clinical chemistry parameters were otherwise unremarkable, except for a small increase of α-amylase activity in plasma. Leptin levels in *Nbea*+/− mice were increased only in proportion to the higher body fat content, and these animals were not leptin-resistant just prior to the onset of the overweight phenotype, as demonstrated by their hypophagia and c-Fos induction in response to leptin administration. At the age of 16 to 18 weeks *Nbea*+/− mice fed *ad libitum* consumed standard chow in greater quantities and exhibited higher daily metabolizable energy intake as compared to WT mice (Table 1), but only in proportion to body mass [22]. Reduced locomotor activity or body temperature as possible explanations of weight gain could not be detected, neither in the primary phenotyping (Table 1) nor in the experiment of Table 2. Rather, also in additional measurements, body temperature always tended to be increased. Taken together, our primary screen data did not reveal an explanation for the development of mild obesity in *Nbea*+/− mice.

Metabolic abnormalities in mildly obese mice, such as *Nbea*+/−, are very small and difficult to detect in whole body energy balance studies. It has been pointed out that the statistical power required to detect a slight but relevant imbalance between energy intake and expenditure is usually not attained with the small cohort sizes used in most animal experimentation [23]. We therefore conducted a second, more detailed analysis of whole body energy balance in a new cohort of mice just before the emergence of increased body fat accumulation. Energy assimilation and energy expenditure were monitored in parallel, but neither parameter as such was significantly altered. However, we found a small but significant elevation of the in-out difference of both parameters in *Nbea*+/− as compared to WT mice. In terms of absolute surplus energy gain the higher in-out-difference of about 3 kJ per day in *Nbea*+/− mice is sufficient to build up excess fat stores of about 1 g over less than two weeks [23].

Notably, an increased drive to consume food was revealed in the *Nbea*+/− mice by presenting them with a defined meal of standard chow following a single period of mild food deprivation. *Nbea*+/− animals ingested significantly more calories than WTs even though no detectable difference in body weight between the mutant and WT animals had as yet developed. Therefore heterozygotes have a higher baseline consumption reflecting their elevated body weight and additionally, under certain food availability conditions, they are more prone to episodic overeating that exceeds the body weight-adjusted control values. In line with this, our data on liquid diet intakes indicate that *Nbea*+/− animals, regardless of their energy status (i.e., hungry or sated), episodically overconsume tastants providing energy, as evidenced by our finding that non-deprived heterozygotes ingest more calorie-containing solutions of sucrose, glucose, fructose or a lipid emulsion (Intralipid) than WTs. An increase is also observed upon *ad libitum* exposure to high-fat high-sugar chow. It is important to note that this elevated intake of solid and liquid diets does not appear to be primarily driven by an increased response to food reward. While palatability serves as a co-stimulus to ingest food, the absence of calories prevents heterozygotes from consuming more than WTs even when the level of feeding reward is high, as *Nbea*+/− and WT mice did not differ in the amount of ingested non-caloric tastants, including palatable saccharin, sucralose and saline.

As Nbea has been shown to be involved in development of and neurotransmission within brain networks [9], we investigated the potential influence of Nbea haploinsufficiency on brain mechanisms pertaining to energy balance. Our gene expression data suggest changes in the activity of the circuitry governing energy homeostasis. Sated *Nbea*+/− animals overexpress orexigenic DYN in the hypothalamus. This may predispose them to eating more upon exposure to caloric foods of desirable characteristics (e.g., palatability, texture). An increased sensitivity of *Nbea*+/− mice to naltrexone in the feeding model strengthens the link between the opioid system and dysregulation of energy balance in Nbea haplosufficiency. DYN, which mediates maintenance of feeding as well as reward [24], [25], is a plausible candidate for such a function. Negative energy balance induced by food deprivation led to increased expression of as many as four hypothalamic genes in *Nbea*+/− compared to WT mice. Two of them, DYN and ORL1, encode orexigens [24], [26], [27]. This suggests an enhanced sensitivity of the hypothalamic feeding circuitry to calorie deprivation in *Nbea*+/− mice, associated with upregulated expression of neuropeptides which stimulate feeding. Since POMC codes for anorexigenic melanocyte stimulating hormone, changes in its expression may also reflect a compensatory mechanism in response to a positive energy balance in the mutant. In contrast to the findings in the deprived and sated state, exposing animals to the palatable diet did not cause differential expression of any gene between *Nbea*+/− and WT mice in the hypothalamus.

We searched HapMap (www.hapmap.org) for SNPs in the human *NBEA* gene with the aim to genotype a frequent SNP as well as a rare SNP that could have a higher penetrance than a common SNP. *NBEA* is a large gene, ∼730 kb, and based on HapMap data the gene contains over 30 haplotype blocks. The two SNPs we selected, rs17775456 and rs7990537, are part of two separate haplotype blocks spanning around 261 and 54 kb, respectively. These SNPs were genotyped in two cohorts: one consisting of severely obese Swedish children and adolescents (mean age 12.6±3.3 years and mean BMI SDS 6.2±1.4) and their age-matched normal-weight controls, and another one of Swedish men born 1920–1924, thus reaching adulthood prior to the appearance of today's obesogenic environment. Individuals in both cohorts showed a significant association of *NBEA* polymorphism with BMI. We found associations for both, rs17775456 and rs7990537, with both body weight and BMI in overweight adult men, while one of the SNPs showed association with BMI in the normal-weight children. In both cohorts, the same allele was associated with high BMI. No genetic variants in *NBEA* have previously been reported to be associated with obesity. However, one of the latest papers on novel loci for BMI [28] estimates that there are at least an additional 300 undiscovered variants that can be linked to obesity. In addition, according to data on 1479 subjects from the British 58 Birth Cohort (www.b58cgene.sgul.ac.uk/index.php) six SNPs in the two haplotype blocks harboring our SNPs are nominal associated with BMI at the age of 44–45 with the same effect direction. This suggests that *NBEA* may be linked to a moderately adipogenic activity which, in children, is better detectable in a normal-weight than in a severely obese background, and only manifests as overweight at an adult age.

We conclude that neural circuitries involved in food intake and body weight control are sensitive to moderate variation of Nbea activity such as haploinsufficiency. Morphological and electrophysiological abnormalities of cortical neurons have indeed been detected in *Nbea*+/− mice [29]. The reduction of Nbea expression by only 50% suffices to cause monogenic adiposity, at least in the mouse model and the C57BL/6N genetic background. This points out human *NBEA* as a potential genetic factor in common, polygenic obesity in collusion with additional genes. Even more subtle variations of NBEA expression, activity or regulation may contribute to polygenic obesity, as a risk or protective factor. The *NBEA* gene is very large (730 kb, 58 exons) and recombination-prone [13], offering extensive mutation potential.

Recent genome-wide association studies emphasize the high proportion of neuronally expressed genes implicated in obesity. Indeed, human obesity has been characterized as “a heritable neurobehavioral disorder that is highly sensitive to environmental conditions” [30]. Our characterization of the obese *Nbea*+/− phenotype is a case in point for both parts of this statement. Nbea is expressed in apparently all neuronal and endocrine cell types [6] and probably has a broad importance for nervous system development and function [9]. In spite of this pleiotropy, the first macroscopic manifestation of Nbea haploinsufficiency to be detected in mice is an impact on body weight, reflecting the subtlety and vulnerability of the neural control of energy balance. Moreover, our findings that the *Nbea*+/− mice are more prone than WT mice to respond to episodic feeding paradigms by overconsuming can be seen as a perturbed ability to handle nutritional challenges, an important factor also in human obesity.

It is intriguing that heterozygous perturbations of the *NBEA* gene have been linked to three dissimilar medical conditions: autism, multiple myeloma, and now obesity. Whereas involvements in autism and obesity may be explained by impacts of NBEA underexpression on the development or functioning of different neuronal circuitries, the association with cancer may be due to functional overlap with its ubiquitous isoform, LRBA [31]. It seems to be a common feature of BEACH proteins that they are involved in the targeting of multiple membrane proteins, and that their KOs therefore generate pleiotropic but partial defects [9]. Autism, cancer and obesity all are typical polygenic disorders, and in combination with different sets of additional risk genes, NBEA misexpression may contribute to different manifestations.

## Materials and Methods

### Animals

Construction and genotyping of the *Nbea* gene-trap KO mice has been described [9]. Analyses described here were performed with animals after backcrossing into the C57Bl/6N background for 5 generations or more. Animal experiments were performed at the animal facilities of Uppsala University or the GMC at the Helmholtz Zentrum München, Germany. All studies received prior approval from the local animal ethics committees and adhered to the German, Swedish and EU laws pertaining to the protection of animals.

### Western blot analysis of Nbea expression

Immunoblots of 5% SDS-polyacrylamide gels were sequentially probed with affinity-purified rabbit sera directed against isoform-specific sequences of mouse Nbea and mouse Lrba generated in our laboratory (S.S. & M.W.K.), and with an anti-pan-cadherin mAb (Sigma C1821). Chemiluminescence-exposed X-ray films were analyzed by densitometry. Whole-brain homogenates were prepared from four animals of each genotype (aged 6 months), adjusted for equal protein concentrations, and dilution series of all four sample sets were analyzed twice by Western blotting and densitometry. Nbea and Lrba signals of each lane were normalized on the respective cadherin control signal.

### Body composition, energy metabolism, and clinical chemistry

Metabolic functions of WT and *Nbea*+/− mice were characterized in a comprehensive systemic phenotyping screen [14], [15]. In total, 140 mice entered the GMC in three cohorts. The primary screen was conducted using the first cohort of 60 mice (n = 15 per sex and genotype for clinical chemistry [tested at 12–13 and 17–18 weeks] and DXA [age, 16–18 weeks]; a subsample of n = 7 mice per sex and genotype in the energy metabolism screen). Groups of up to four mice per cage were housed on a 12-h light/dark cycle, had *ad libitum* access to regular laboratory chow (Altromin1324; Altromin, Lage, Germany), and were provided with UV-irradiated and micro-filtered tap water. For energy assimilation monitoring (age 18–20 weeks), mice were single-caged on grid panels (0.5-cm grid hole diameter) allowing the collection of feces and spilled food of individual mice. Body weight, food consumption, mean rectal body temperature of five consecutive measurements conducted at 10 a.m. every day, daily feces production calculated from a five-day pooled sample, energy uptake, energy content of the feces, metabolizable energy and the food assimilation coefficient were determined. Samples of the lab chow and feces (∼1 g) were dried at 60°C for two days, homogenized in a grinder and squeezed to a pill for determination of energy content in a bomb calorimeter (IKA Calorimeter C7000). Energy uptake was determined as the product of food consumed and the caloric value of the food. To obtain metabolizable energy, the energy content of feces and urine (2% of energy uptake) was subtracted from energy uptake. Two-way ANOVA (SigmaStat, Jandel Scientific) was used to test for effects of the factors, genotype and sex. To adjust for body mass differences in energy metabolism parameters, a linear regression model was applied including body mass as a co-variate (TIBCO Spotfire S+ 8.1 for Windows). A pDEXA Sabre X-ray Bone Densitometer (Norland Medical Systems Inc., Basingstoke, Hampshire, UK) was used for dual-energy X-ray absorptiometry (DXA). The entire body area including and excluding the skull was assayed with a 0.02-g/cm^2^ Histogram Averaging Width (HAW) setting.

In the second cohort of mice (n = 10 per sex and genotype), body composition was followed up by non-invasive qNMR scans (MiniSpec LF60, Bruker Optics, Germany). For the evaluation of energy balance, single mice were kept in respirometry cages (Phenomaster System, TSE Systems, Germany) including the monitoring of gas exchange, food and water uptake, and locomotor activity. To convert food consumption into caloric uptake, energy extraction efficiency of individual mice was determined as described above. Li-heparin plasma samples for clinical chemistry analyses were obtained by blood collection from the retroorbital vein plexus of ether-(first cohort) or isoflurane-anesthetized mice into heparinized tubes. Samples were mixed thoroughly and stored for 2 h at room temperature before being separated from blood cells by centrifugation (4656× g; 10 min). Plasma samples were stored at 4°C and analyzed within 24 h using an AU400 autoanalyzer (Olympus, Hamburg, Germany) and adapted test kits from Olympus. Adipokine determinations were performed with the LINCOplex mouse serum adipokine multiplex immunoassay kit MADPK-71K-07. Two-way ANOVA (SigmaStat, Jandel Scientific) was used to test for effects of genotype and sex, and significance of mean differences between genotypes within each sex was tested using the Welsh t-test (Excel, Microsoft).

### HF feeding and glucose tolerance test

Beginning at the age of 14 weeks, a third cohort of mice (n = 10 per sex and genotype) were fed a HF diet (D12492, Research Diets, New Brunswick NJ, USA) for further 24 weeks. 60% of the total energy content (23.2 kJ g^−1^) was due to fat (lard and soybean oil). Mice were weighed every week. After 22 weeks, an intraperitoneal glucose tolerance test was conducted after overnight fasting according to the EMPReSSslim protocol (www.eumodic.eu). Blood samples were taken from the tail vein prior, 15, 30, 60, 90, and 120 minutes after intraperitoneal injection of the 2 mg per g body mass glucose bolus.

### Episodic feeding experiments

Animals were housed individually in a temperature- (21–23°C) and humidity-controlled facility with a 12:12 LD cycle (lights on at 06:00). Age-matched (±2 days) males were used simultaneously. Tap water and standard chow (Lactamin, Lidköping, Sweden) were available *ad libitum* unless specified otherwise. Sixteen age-matched animals of each genotype were used in Experiments 1, 2 and 3, whereas 11 mice per genotype were included in Experiment 4. Mice were between the 8^th^ and 10^th^ week of age, before the emergence of a statistically significant difference in body weight between the genotypes: 18.0±0.4 g in heterozygotes and 17.5±0.3 g in WT controls.

#### Experiment 1: Effect of genotype on ad libitum consumption of the energy-dense and palatable HFHS (high-fat high-sugar) diet

Standard chow was removed from cages at 09:00 and it was replaced with the palatable HFHS pellets (Research Diets). During the first 24 h of exposure to the HFHS food, the animals were allowed to get accustomed to the new diet. Then the amount of ingested food (corrected for spillage) was measured for the period of 24–72 h.

#### Experiment 2: Effect of genotype on food deprivation-induced re-feeding

Standard chow was removed just before the onset of darkness and it was returned at 10:00 on the subsequent day. The amount of ingested food (corrected for spillage) was measured 2 h later when the major consummatory activity had subsided.

#### Experiment 3: Effect of genotype on ad libitum consumption of caloric and palatable Intralipid and sugar solutions

Mice were given a bottle of 10% sucrose, 4.1% Intralipid, 10% glucose or 10% fructose in addition to chow for 48 h. Intralipid (Fresenius, Sweden), a palatable lipid emulsion of soybean oil, glycerol and egg yolk phospholipids, has been used in experiments utilizing liquid diets [32]. Sucrose, glucose, fructose and Intralipid were isocaloric (0.4 kcal/g), whereas the energy content of chow was 3.6 kcal/g. During the first 24 h of exposure to the liquids, the animals were allowed to get accustomed to the new tastants. Uptake was measured during the following 24 h.

#### Experiment 4: Effect of genotype on ad libitum consumption of non-caloric but palatable saccharin, sucralose, and saline solutions

Mice were given a bottle of 0.1% saccharin, 0.05% sucralose or saline in addition to chow for 48 h. Total caloric intake per mouse in the saccharin paradigm in heterozygotes was 12.53±0.7 kcal and in WTs, 13.39±0.5 kcal; in the sucralose paradigm, heterozygotes ingested 14.89±0.7 kcal and WTs, 14.53±0.5 kcal; in the saline paradigm, heterozygotes ingested 13.19±0.4 kcal and WTs, 13.25±0.5 kcal. During the first 24 h of exposure to the liquids, the animals were allowed to get accustomed to the new tastants.

#### Experiment 5: Effect of leptin administration on deprivation-induced food intake by *Nbea+/−* mice

Chow was removed from the hoppers of *Nbea*+/− mice before the onset of darkness. On the next day, at 11:00, the animals were injected i.p. with 10 µg leptin or vehicle (n = 5/group) and the pre-weighed food pellets were returned to the hopper. Food intake was measured 3 h post-injections and corrected for spillage.

#### Experiment 6: Effect of naltrexone (NTX) on deprivation-induced food intake

Chow was removed from the hoppers of *Nbea*+/− and WT mice before the onset of darkness. On the next day, at 11:00, the animals were injected i.p. with 3 mg/kg NTX or vehicle (n = 5–7/group) and the pre-weighed food pellets were returned to the hopper. Food intake was measured 3 h post-injections and corrected for spillage.

### Real-time PCR analysis of expression of feeding-related genes

#### Hypothalamic and brainstem gene expression after 16-h food deprivation

Standard chow was removed just before the onset of darkness and mice (n = 8/genotype) were decapitated between 10:00 and 11:00 on the next day. The fed controls (n = 8/genotype) had food *ad libitum* and were decapitated between 09:00 and 10:00. mRNA expression in the hypothalamus and brainstem was studied with real-time PCR.

#### Hypothalamic Nbea expression following 48-h consumption of the HFHS diet

Mice that had been pre-exposed 5 days earlier to the HFHS food (Research Diets) for 1 day were given this diet again for 48 h; controls (n = 8/genotype) had standard chow only. Mice were decapitated after 48 h (between 10:00 and 11:00). mRNA expression in the hypothalamus was studied with real-time PCR.

#### PCR methodology

Brains were excised, anatomical regions of interest dissected, immersed in RNAlater (Ambion, USA), kept at room temperature for 2 h and, thereafter, stored at −80°C until further processed. Samples were homogenized and RNA prepared as previously described [33]. Relative expression levels of seven housekeeping genes (glyceraldehyde-3-phosphate-dehydrogenase, β-tubulin, ribosomal protein 19, histone H3, cyclophilin, β-actin, and succinate dehydrogenase complex subunit B) and of the genes of interest were determined with quantitative rtPCR. Each reaction, with a total volume of 20 µl, contained 20 mM Tris/HCl pH 9.0, 50 mM KCl, 4 mM MgCl_2_, 0.2 mM dNTP, DMSO (1∶20) and SYBR Green (1∶50000). Template concentration was 5 ng/µl and the concentration of each primer was 2 pmol/µl. Primers were designed with Beacon Designer (Premier Biosoft) using the SYBR Green settings. All rtPCR experiments were performed in duplicates; for each primer pair a negative control with water and a positive control with 5 ng/µl of genomic DNA were included on each plate. Amplifications were performed with 0.02 µg/ml Taq DNA polymerase (Biotools, Sweden) under the following conditions: initial denaturation at 95°C for 3 min, 50 cycles of denaturing at 95°C for 15 sec, annealing at 52.8–60.1°C for 15 sec and extension at 72°C for 30 sec. Analysis of rtPCR data was performed as previously reported [34]. MyIQ 1.0 software (Bio-Rad) was used. Primer efficiencies were calculated using LinRegPCR [35] and samples were corrected for differences in primer efficiencies. The GeNorm protocol described by Vandesompele et al. [36] was used to calculate normalization factors from the expression levels of the housekeeping genes. Grubbs' test was used to remove outliers. Differences in gene expression between groups were analyzed with ANOVA followed by Fisher's PLSD test where appropriate. P<0.05 was used as the criterion of statistical significance.

### Human genetic studies

#### Subjects

We genotyped 1152 men from the Uppsala Longitudinal Study of Adult Men (ULSAM), described previously [37]–[38]. The study was approved by the Ethics Committee of the Department of Medicine, Uppsala University. All participants gave their written informed consent. We further genotyped 1027 children and adolescents comprising one group of obese children and adolescents and one group of normal-weight adolescents, as described earlier [39]. The study was approved by the Regional Ethics Committee in Stockholm.

#### Phenotype characterization

For the men, height was measured to the nearest whole centimeter and weight to the nearest whole kilogram at age 50 years. BMI was calculated as weight divided by height squared (kg/m^2^). For the children and adolescents, body weight and height were measured to the nearest 0.1 kg and 1 cm, respectively. BMI standard deviation score (BMI SDS) was calculated from weight and height and standardized for age and sex [40].

#### Genotyping

Genotyping of *NBEA* rs7990537 and rs17775456 variants in the ULSAM cohort was carried out at the SNP technology platform at Uppsala University (http://www.genotyping.SE/) using an Illumina Golden Gate Assay [41]. The SNP genotype call rate in the samples was 96.8%. For the children and adolescents, genomic DNA was extracted from peripheral blood using QiaGen Maxiprep kit (Qiagen, Hilden, Germany). Genotyping was performed with pre-designed Taqman single-nucleotide polymorphism genotyping assay (Applied Biosystems, Foster City, USA) and an ABI7900 genetic analyzer with SDS 2.2 software.

#### Statistical analysis

In order to test for deviation from Hardy-Weinberg equilibrium, the Person's χ^2^-test (1 d.f) was applied. Association analysis of rs17775456 and rs7990537 with obesity measurements was performed using BMI and body weight as continuous traits. The adult men were categorized as overweight (BMI≥25 kg/m^2^) or normal-weight (BMI<25 kg/m^2^) before the analysis. Skewed variables were log-transformed and analyzed with linear regression, assuming an additive model. Covariates such as height, age and sex were tested for dependence on the response variables and included in the model if significant. In order to correct for multiple comparisons Bonferroni correction was applied and a p-value≤0.006 was considered statistically significant. All the analyses were performed using PLINK (http://pngu.mgh.harvard.edu/purcell/plink/) [42].

#### Power analysis

Power calculation was carried out with the CaTS power calculator (www.sph.umich.edu/csg/abecasis/CaTS/index.html) [43] and Power and Sample Size Calculation (biostat.mc.vanderbilt.edu/wiki/Main/PowerSampleSize) [44]. For rs17775456 we had 80% power to detect association with overweight and obesity, with a relative risk of 1.2. For rs7990537 we had 80% power to detect association with overweight and obesity with a relative risk of 1.4.

## Supporting Information

Figure S1 ARC neurons of *Nbea*+/− mice are sensitive to leptin. A: An intraperitoneal injection of leptin at the dose that reduces food intake causes an increase in the density of Fos immunoreactive (IR) nuclei in the ARC of WT and *Nbea*+/− mice (established bilaterally per mm^2^ of the tissue on every fourth ARC section). One animal per genotype was injected with saline or leptin (treatment details as in the feeding experiment). An hour later the mice were perfused transcardially with 4% paraformaldehyde. Coronal brain sections (50 µm) were cut on a vibratome and immunostained. Images provided by the camera attached to the Nikon microscope were analyzed using the NIH 1.51 Image software (NIH, MD). Full description of Fos staining in [32]. B: Photomicrographs depicting c-Fos staining in the ARC of saline- (left) versus leptin-treated (right) *Nbea*+/− mice. VMH, ventromedial hypothalamic nucleus; 3v, 3^rd^ ventricle.
(TIF)
