# Supplementary figures and images for: Neurobeachin, a Regulator of Synaptic Protein Targeting, Is Associated with Body Fat Mass and Feeding Behavior in Mice and Body-Mass Index in Humans
Source: PLoS Genet. 2012 Mar 15;8(3):e1002568. doi: 10.1371/journal.pgen.1002568 (PMC3305408; doi:10.1371/journal.pgen.1002568)

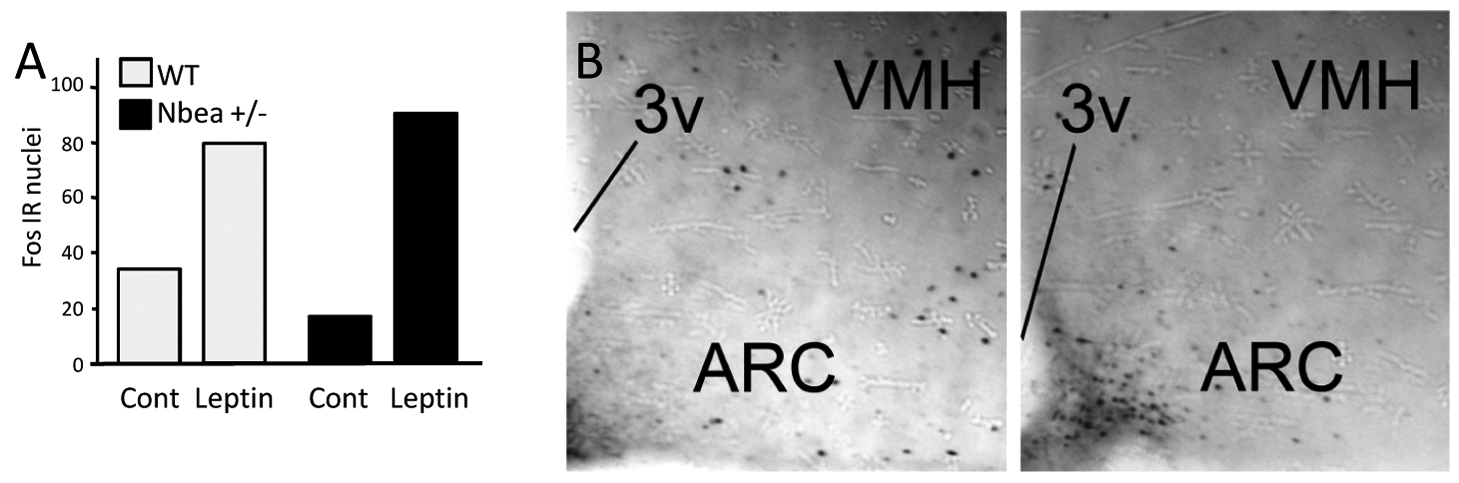

Supplement: Figure S1 — ARC neurons of Nbea+/− mice are sensitive to leptin. A: An intraperitoneal injection of leptin at the dose that reduces food intake causes an increase in the density of Fos immunoreactive (IR) nuclei in the ARC of WT and Nbea+/− mice (established bilaterally per mm2 of the tissue on every fourth ARC section). One animal per genotype was injected with saline or leptin (treatment details as in the feeding experiment). An hour later the mice were perfused transcardially with 4% paraformaldehyde. Coronal brain sections (50 µm) were cut on a vibratome and immunostained. Images provided by the camera attached to the Nikon microscope were analyzed using the NIH 1.51 Image software (NIH, MD). Full description of Fos staining in [32]. B: Photomicrographs depicting c-Fos staining in the ARC of saline- (left) versus leptin-treated (right) Nbea+/− mice. VMH, ventromedial hypothalamic nucleus; 3v, 3rd ventricle. (TIF) [file pgen.1002568.s001.tif]
